# Supplementary material for: Comprehensive Annotation and Functional Exploration of MicroRNAs in Lettuce
Source: Front Plant Sci. 2021 Dec 24;12:781836. doi: 10.3389/fpls.2021.781836 (PMC8739914; doi:10.3389/fpls.2021.781836)
Supplement: Supplementary file 2 [file Data_Sheet_8.ZIP › GSM2230750.plot/Lsa-miR159b_Lsat_1_v5_gn_5_75800.1_1860_TPlot.pdf]

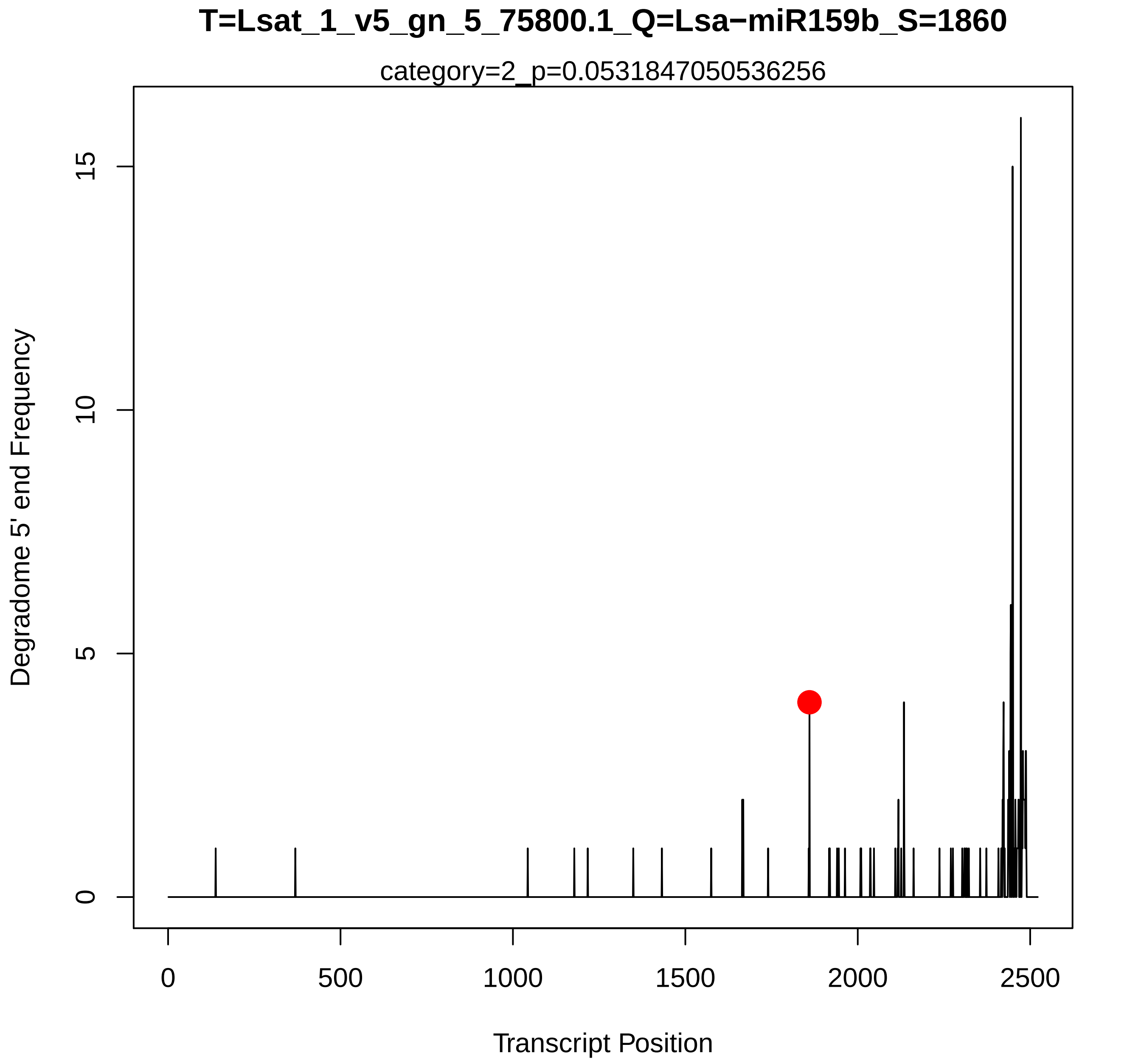

T=Lsat_1_v5_gn_5_75800.1_Q=Lsa−miR159b_S=1860
category=2_p=0.0531847050536256
Degradome 5' end Frequency
0
5
10
15
0
500
1000
1500
2000
2500
Transcript Position
